# The Brief Form of the California Odor Learning Test 3

**DOI:** 10.3389/fnins.2020.00173

**Published:** 2020-03-24

**Authors:** Conner Frank, Claire Murphy

**Affiliations:** ^1^Department of Psychology, San Diego State University, San Diego, CA, United States; ^2^Department of Psychiatry, University of California, San Diego, San Diego, CA, United States

**Keywords:** odor memory, odor learning, aging, olfaction, neuropsychological assessment

## Abstract

This study explored whether a Brief Form of the California Odor Learning Test 3 (COLT), an olfactory analog of the newly released Brief Form of the California Verbal Learning Test (CVLT 3), could retain the ability of the COLT to detect odor memory dysfunctions observed in normal aging. 52 participants, 28 young (18–30 years old) and 24 old (65 years of age and older), were administered the Brief Forms of the CVLT 3 and the COLT 3. Results indicated poorer performance in immediate and delayed odor recall in older than in younger adults. Poorer odor recognition memory performance in older adults than in younger adults was detected. This study suggests that the Brief Form of the COLT can detect differential odor learning and memory between young and older adults. Thus, the current brief test holds promise as a measure that can be incorporated into studies that demand a brief, non-invasive test capable of detecting impairment in olfactory function.

## Introduction

Alzheimer’s Disease (AD) is a terminal neurodegenerative illness that causes debilitating deficits in memory and cognition. Previous studies estimate that 5–6% of adults over 65 develop dementia, and 50–80% of these cases can be attributed to AD (Eibenstein et al., 2005b). The AD disease process is estimated to last 15 to 24 years; however, the large majority of the AD disease process is preclinical. While the disease process is highly variable, clinical manifestations are estimated to only be detectable in the late stages of the disease (Vermunt et al., 2019). Since the pathological process of AD occurs more than a decade prior to the clinical stage of the disease, by the time AD is diagnosed, patients have already experienced a decade of significant neuronal damage, contributing to the lack of success in developing successful treatments for AD (Sperling et al., 2011, 2014). Thus, there is a critical need to determine a process for identifying and studying individuals at high risk of developing clinical manifestations of the AD prior to the onset of irreversible neuropathology.

A promising avenue for detection of risk for AD and insipient preclinical neuropathology is through the sense of smell (Murphy, 2019). The prevalence of olfactory dysfunction increases with age, with 24.5% of adults over the age of 50 and 62.5% of adults over 80 experiencing deficits (Murphy et al., 2002). Olfactory memory abilities can be split into several domains, including odor identification, or the ability to attach a correct verbal label to an odor; odor recognition, or the ability to recognize a previously presented odor; and odor recall, or the ability to retrieve an episodic memory of an odor. Older adults have been found to be impaired on many aspects of olfaction, including immediate odor recall, delayed and cued odor recall, odor labeling, and odor recognition, even when compared to verbal memory (Doty et al., 1984; Russell et al., 1993; Murphy et al., 1997; Nordin and Murphy, 1998; Laakso et al., 2009). These deficits cannot be attributed to poor odor detection alone, and have been found to be more related to semantic and verbal processing and encoding (Murphy et al., 1991; Larsson and Bäckman, 1993; Larsson and Bäckman, 1997; Lehrner, 1999). This supports the assertion that olfactory deficits observed in old age are more associated with deficits in higher cognitive functioning. In addition, the earliest brain degeneration in AD is observed in brain areas linked to olfaction and memory, such as the perirhinal cortex, anterior olfactory cortex, entorhinal cortex, and the anterior parahippocampal gyrus (Braak and Braak, 1991; Price et al., 1991). Studies that investigate the neural organization of the olfactory, limbic, and medial temporal lobes offer additional support, as the olfactory nerve is separated by only two synapses from the amygdala and three synapses from the hippocampus (Hyman et al., 1991), and hippocampal lesions have been found to severely impact odor place associations (Goodrich-Hunsaker et al., 2009). Therefore, there is significant evidence that the olfactory system shares an intimate link with memory systems and that deficits in olfaction can be effectively used as a measure of semantic and verbal processing abilities.

Because of this intimate connection between olfactory processing and medial temporal lobe functioning, olfaction has been heavily implicated as a predictor of AD conversion. One of the most promising signs of a relationship between preclinical AD and olfaction is the observation of AD related pathology, including amyloid plaques and neurofibrillary tangles, in regions of the brain important for olfactory processing (Braak and Braak, 1991; Christen-Zaech et al., 2003). In addition, medial temporal lobe areas important in memory and cognition have been associated with odor recall and recognition deficits in lesion studies (Rausch et al., 1977; Eibenstein et al., 2005a). Decreases in odor identification and odor memory have also been associated with decreases in volume of the hippocampus, decreases in entorhinal cortex thickness, and worse episodic memory (Murphy et al., 2003; Growdon et al., 2015; Dhilla Albers et al., 2016). As expected based on these observations, deficits in odor identification have been observed to be associated with mild cognitive impairment (Eibenstein et al., 2005b), conversion from mild cognitive impairment to AD (Devanand et al., 2000; Roberts et al., 2016), and with patients diagnosed with probable or questionable AD (Kesslak et al., 1988; Serby et al., 1991; Morgan et al., 1995). In addition, deficits in odor recall, recognition, and familiarity have been found to be associated with mild cognitive impairment and AD (Nordin and Murphy, 1998; Niccoli-Waller et al., 1999; Dhilla Albers et al., 2016). Deficits in odor identification and memory have also been observed to be associated with the genetic risk factor for AD, the apolipoprotein e4 (APOE) allele (Murphy et al., 1998; Gilbert and Murphy, 2004a, b; Calhoun-Haney and Murphy, 2005; Handley et al., 2006; Olofsson et al., 2010, 2020). In contrast, deficits in odor detection have been found to appear later into AD pathology (Serby et al., 1991; Bacon et al., 1998; Murphy, 1999). Thus, there is significant evidence that higher cortical functions, such as identification, recall, and recognition, are associated with preclinical AD pathology.

However, more research needs to be conducted to create an olfactory assessment that successfully predicts preclinical AD with necessary sensitivity and specificity. With this goal in mind, this study focused on creating a brief version of the California Odor Learning Test (COLT), an odor memory test developed by Murphy et al., in 1997. The original form of the COLT was created as an olfactory analog to the California Verbal Learning Test (CVLT), a verbal learning test developed by Delis et al. (1987). The CVLT is a useful assessment for measuring rates of verbal learning; recall, or the ability to retrieve verbal labels previously presented; and recognition memory, or the ability to recognize previously presented verbal labels. It has been previously found that MCI patients and AD patients display reduced learning rates, rapid forgetting, increased intrusion errors, and poor recognition abilities with increased false positives on the CVLT (Greenaway et al., 2006; Campos-Magdaleno et al., 2017). The COLT is a measure of odor learning and recall, both immediate and delayed, odor intrusion errors, odor recognition, and odor identification, and has been found to be sensitive to aging and neurodegeneration (Murphy et al., 1997; Hamilton et al., 1999; Murphy, 2019). Thus, this study aimed to create a brief version of the COLT to be administered in a standardized battery of neuropsychological assessments that retained the sensitivity to olfactory degeneration in normal aging. We reasoned that creating a test that retained the COLT’s sensitivity to aging was an important first step in developing a brief version of the COLT that had the potential for inclusion in future studies in preclinical AD. Such a test may allow for better detection of precipitating pathological changes in AD through inclusion of olfactory assessment in clinical assessment and clinical trials. The current study sought to compare performance between cognitively normal older adults and younger adults on a brief version of both the CVLT and COLT, with the intention of developing a test that can be used in the future to aid in assessment for preclinical AD.

## Materials and Methods

### Participants

Twenty eight young (18–30 years old) and 24 older adults 65 years or older) participated in this research. Younger participants were 19 females, 9 males, with a mean age of 19.17 years (SD = 0.87) and a mean education level of 13.04 years (SD = 1.20). Older participants were 13 females, 11 males, with a mean age of 74.29 (SD = 6.94) and a mean education level of 16.54 years (SD = 3.80). Older participants were given the MMSE to screen for signs of dementia, and all participants attained a score of 25 or greater (*M* = 27.87, SD = 1.46). All participants gave informed consent in writing and were compensated for participation.

### Materials and Procedures

Subjects were given a battery of neuropsychological assessments composed of the CVLT 3 Brief Form (Delis et al., 2017) and the COLT 3 Brief Form. The MMSE (Folstein et al., 1975) was given to rule out dementia in older participants, and half the younger adults also had scores for the test. Though the focus in this study was on the Brief form of the COLT, 20 of 24 old and 12 of 28 young participants had also taken the San Diego Odor Identification Test (Murphy et al., 2002). Further, odor identification abilities were assessed using the odor identification portion of the Brief Form of the COLT 3.

### Brief Form of the CVLT 3

Subjects were administered the Brief Form of the CVLT 3, a short form of the CVLT released in the 3rd edition of the CVLT (Delis et al., 2017). The CVLT 3 is a verbal learning and memory test designed to test immediate recall, short delay recall, long delay recall, and recognition memory, as well as the memory strategies being used by the participant. The Brief Form of the CVLT 3 consists of nine words, administered repeatedly over 4 immediate recall trials. Following the immediate recall trials, the Brief Form has a distractor task in which participants are asked to count backward from 100 for 30 s. Following this task, participants complete the short delay free recall task. There is no short delay cued recall task in the Brief Form of the CVLT 3. Next there is a 10-min break, followed by a long delay recall section, including both free and cued recall. Finally, there is a yes/no verbal recognition task, in which 27 items are presented, 18 of which had not been previously presented.

### Brief Form of the COLT 3

An olfactory analog to the Brief Form of the CVLT 3 was created. This test was identical to the CVLT 3 Brief Form in procedure, but differed in that it contained odors instead of words. The procedure was as follows; subjects were administered 9 common household odors (e.g., coffee) in each of the three categories over 4 immediate recall trials. Odors were administered for 5 s each with an inter-stimulus interval of 10 s in order to replicate the procedure used in previous studies with the COLT (Murphy et al., 1997). Subjects then completed a distractor task identical to the distractor task in the CVLT 3 Brief Form consisting of counting backward from 100 for 30 s. Next, subjects completed the short delay free recall tasks, followed by a 10-min delay interval, followed by the long delay free and cued recall tasks. On recall tasks, responses were scored as repetitions if a participant listed an odor more than once and scored as an intrusion if the participant listed an odor not presented. Subjects completed a yes/no recognition task that consisted of 27 odors, composed of the 9 odors from the immediate recall section along with 18 odors not previously presented. Finally, subjects completed an odor identification task, the only other difference from the format of the CVLT 3 Brief Form. In this task, the 9 odors from the immediate recall were presented to participants, one at a time, and participants were asked to give a verbal label for their best guess as to the identity of the presented odor. These responses were not principally used to assess odor identification abilities, however. This information was used to create an additional scoring procedure for the immediate and delayed recall portions of the test. When participants reported a verbal label for an odor that differed from the actual name of the odor, the accurate label of the odor was replaced and subjects were instead rated on the number of times they responded with the verbal label reported in the identification section, in order to preserve accurate results for participants who inaccurately identified a stimulus but accurately recalled it over several trials.

### Analysis

Analyses of Variance with repeated measures were used to compare performance across age group (young, old) and test (BCVLT-3, BCOLT-3), with separate analyses for immediate recall measures, delayed recall measures and recognition measures. Significant interactions were followed-up with paired comparisons. Effect sizes are given to aid in evaluation of results. Because of the number of statistical tests conducted, an alpha level of 0.01 was chosen to represent a statistically significant difference.

## Results

### MMSE

All old adults were given the MMSE to rule out dementia. Fourteen of the 28 young subjects also had the test and scores were compared between age groups using independent samples *t*-test. Scores were significantly lower for older adults [*t*(38) = 3.68, *p* = 0.014].

### Odor Identification

Though the focus of this study was on the Brief Form of the COLT 3, many of the participants had also taken the San Diego Odor Identification test. These SDOIT scores in 20 of 24 old and 12 of 28 young participants were compared between age groups using a one-way ANOVA. Scores were significantly higher for younger than for older adults [*F*(1,26) = 8.14, *p* = 0.008]. Odor identification scores from the Brief form of the COLT 3 were significantly higher in younger than older adults, [*F*(1, 49) = 9.09, *p* = 0.004], as shown in Table 1.

**TABLE 1:** Means and standard errors for young and old.

Gender (M/F)	Young (9/19)		Old (11/13)	
	Mean	SE	Mean	SE
Age	19.50	0.22	74.29	1.42
BCVLT – Trial 1	5.93	0.19	5.00	0.31
BCVLT – Trial 4	8.25	0.15	7.42	0.23
BCVLT – SDFR	7.96	0.21	6.67	0.34
BCVLT – LDFR	8.00	0.17	6.08	0.39
BCVLT – LDCR	8.00	0.21	6.67	0.38
BCVLT – Recognition	26.71	0.43	25.42	0.38
BCVLT – False Positives	0.32	0.15	0.71	0.28
BCVLT – Intrusions	0.50	0.23	2.00	0.51
BCOLT – Trial 1	4.50	0.31	3.54	0.36
BCOLT – Trial 4	6.50	0.28	4.17	0.37
BCOLT – SDFR	6.79	0.25	4.64	0.31
BCOLT – LDFR	6.50	0.22	4.50	0.31
BCOLT – LDCR	5.96	0.39	4.29	0.39
BCOLT – Recognition	22.71	0.36	21.08	0.63
BCOLT – False Positives	3.29	0.31	4.88	0.57
BCOLT – Intrusions	4.46	0.78	10.67	1.88
BCOLT – Odor ID	3.68	0.27	2.29	0.36
SDOIT (12 Young, 16 Old)	6.25	0.33	3.94	0.66
MMSE (14 Young, 24 Old)	29.36	0.20	27.83	0.29

*SDFR, short delay free recall; LDFR, long delay free recall; LDCR, long delay cued recall; Recognition, total recognition correct.*

### Immediate Recall Measures

**FIGURE 1:**
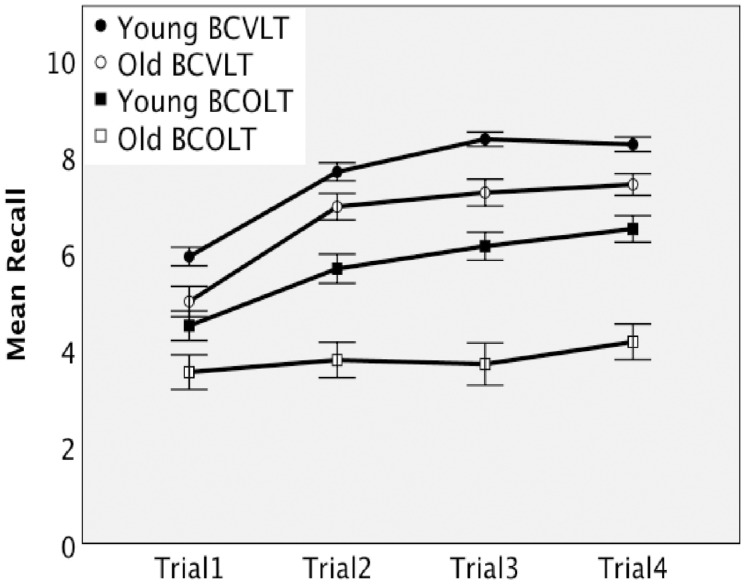
Immediate recall by young and older adults on the Brief Forms of the CVLT and the COLT.

Immediate recall measures were analyzed using a 2 × 2 × 2 ANOVA (age group × test × trial), with repeated measures on test and trial (Trial 1, Trial 4). The mean and standard error scores on the Brief forms of the CVLT 3 (BCOLT3) and COLT 3 (BCVLT3) for the two age groups and trials are displayed in Table 1. Figure 1 shows the difference in recall rates over the immediate recall measures for each age group. Performance on immediate recall was significantly better in young than in older adults and significantly better for words than for odors. The ANOVA revealed significant main effects of trial [*F*(1,50) = 193.47, *p* < 0.001, eta-squared = 0.80], test [*F*(1,50) = 76.84, *p* < 0.001, eta-squared = 0.61], and age group [*F*(1,50) = 23.75, *p* < 0.001, eta-squared = 0.32]. The interaction effects of trial × age group [*F*(1,50) = 5.85, *p* = 0.019, eta-squared = 0.11] and test × age group [*F*(1,50) = 2.89, *p* = 0.095, eta-squared = 0.055] were not significant. The interaction of trials × test was significant, [*F*(1,50) = 14.28, *p* < 0.001, eta-squared = 0.22]. The trial × test × age group interaction was not significant when corrected to 0.01 but approached significance at 0.011, [*F*(1,50) = 6.91, *p* = 0.011, eta-squared = 0.12]. Thus, pairwise comparisons were used to follow up. Both young and older adults showed an increase in recall from trial 1 to trial 4 on the BCVLT3. Older adults showed no increase in recall from trial 1 to trial 4 on the BCOLT3. In contrast, young adults showed a significant increase in recall from trial 1 to trial 4 in the BCOLT3. Young adults outperformed older adults on BCOLT3 trial 4 but not trial 1. Together these comparisons suggest an age-associated difference in odor learning.

**FIGURE 2:**
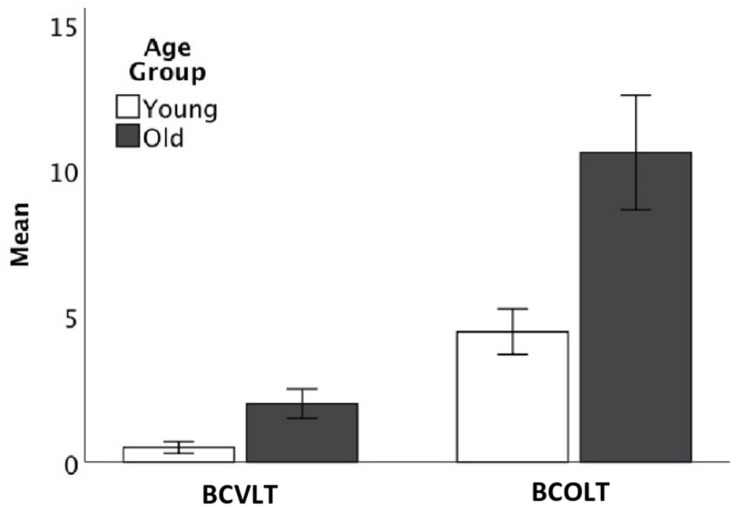
Intrusion errors by young and older adults on the Brief Forms of the CVLT and the COLT.

ANOVA (age group × test) on intrusions showed significant differences for young and old [*F*(1,102) = 14.07, *p* > 0.001, eta-squared = 0.22] and for the BCOLT3 and BCVLT3, [*F*(1,102) = 37.96, *p* > 0.001, eta-squared = 0.44]. The interaction of age group × test approached but was not significant, [*F*(1,102) = 5.18, *p* = 0.027, eta-squared = 0.10]. As shown in Table 1 and Figure 2, older adults made more intrusion errors for both words and odors than younger adults and both younger and older adults made more intrusion errors for odors than for words.

### Delayed Recall Measures

**FIGURE 3:**
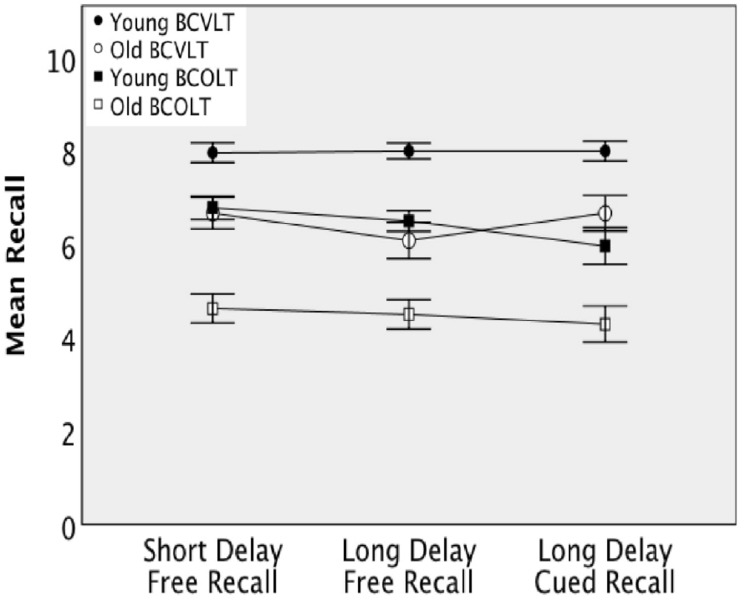
Delayed recall by young and older adults on the Brief Forms of the CVLT and the COLT.

Delayed recall measures were analyzed using repeated measures 2 × 2 ANOVA (age group **×** test). Mean differences between age groups in recall for the delayed recall measures, as well as total mean recall, are displayed for both tests in Figure 3. In addition, mean and standard errors are displayed in Table 1. Performance on delayed recall was significantly better in young than in older adults and significantly better for words than for odors. Responses on the short delay free recall were found to be significantly different between tests [*F*(1,50) = 51.92, *p* < 0.001, eta-squared = 0.51] and age groups [*F*(1,50) = 29.25, *p* < 0.001, eta-squared = 0.37] but the test **×** age group interaction was not significant, [*F*(1, 50) = 3.73, *p* = 0.059, eta-squared = 0.069]. Responses on the long delay free recall were found to be significantly different between tests [*F*(1,50) = 38.68, *p* < 0.001, eta-squared = 0.44] and age groups [*F*(1,50) = 42.21, *p* < 0.001, eta-squared = 0.46], but no significant test **×** age group interaction was observed [*F*(1,50) = 0.028, *p* = 0.87, eta-squared = 0.001]. Similarly, responses on the long delay cued recall were found to be significantly different between conditions [*F*(1,50) = 55.48, *p* < 0.001, eta-squared = 0.53] and age groups [*F*(1,50) = 14.60, *p* < 0.001, eta-squared = 0.23], but no significant test **×** age group interaction was observed [*F*(1,50) = 0.33, *p* = 0.57, eta-squared = 0.007].

### Recognition Measures

Performance on recognition was significantly better in young than in older adults and significantly better for words than for odors. Recognition performances were scored for hits (correctly saying they had smelled an odor previously) and false positives (incorrectly identifying an odor as having been presented earlier when it had, in fact, not been presented). Mean and standard errors for recognition totals, hits, false positives, for both age groups can be seen for both tests in Table 1. Separate 2 × 2 (age group **×** test) repeated measures. ANOVAs were employed for total correct recognition responses, and false positive recognition. A significant main effect of test on total recognition correct [*F*(1,50) = 109.09, *p* < 0.001, eta-squared = 0.69], as well as a main effect of age group [*F*(1,50) = 8.62, *p* = 0.005, eta-squared = 0.15] were observed. No significant test **×** age group interaction was observed for total recognition correct [*F*(1,50) = 0.18, *p* = 0.68, eta-squared = 0.003]. For false positives, a significant main effect of test [*F*(1,50) = 139.05, *p* < 0.001, eta-squared = 0.74] was observed. The main effect of age group [*F*(1,50) = 6.67, *p* = 0.013, eta-squared = 0.12] approached significance. The test **×** age group interaction effect was not significant, [*F*(1,50) = 3.95, *p* = 0.052, eta-squared = 0.073].

## Discussion

The findings in this study provide further evidence that odor recall rates are significantly impaired in normal aging, as reported by Murphy et al. (1997). In fact, the provides significant evidence that the Brief Form of the COLT 3 displays the ability to detect age-associated differences in odor memory.

While older adults did show lower odor identification abilities than young adults, this cannot fully explain the deficits observed in odor learning over the immediate recall trials. Odor identification was controlled for by allowing participants to verbally label the odors they smelled at the end of the test. Because an odor that is consistently recalled with the same incorrect label throughout the test implies something systematic that is not true of an odor that is inconsistently mislabeled on recall, we did a separate analysis to assess recall ability, relatively independent of correct labeling. We assumed that a (mis)label given during odor identification had been used for encoding and thus scored it as correct when it was recalled. These labels were then used to control for odor identification in the recall portion of the tests; i.e., the verbal label used to identify each odor at the end of the test was counted as a correct recall if it was used during the Brief COLT 3. This was done to isolate the rates of odor learning in the absence of lower performance on odor identification. Lower rates of odor learning in the older adults relative to the young adults persisted strongly and replicated previous findings of age related differences in recall acquisition rates specifically for odors on the original COLT (Murphy et al., 1997), creating a strong argument for both the validity of the Brief COLT 3 as a measure of odor learning and age-related lower performance in learning for odors as a major change observed in the aging process.

The delayed recall portion of the test showed a similar pattern. Older adults showed significantly lower performance in both modalities when compared to young adults. Both young and old adults performed significantly lower on delayed recall for odors as opposed to words. Unsurprisingly, a similar pattern emerged in the delayed recall as in the immediate recall. Older adults displayed lower performance over the delayed recall trials for odors, and younger adults also displayed lower performance for odors. This suggests that, in general, delayed recall shows a pattern of poor recall for odors (Figure 3).

Recognition memory for odors was significantly lower than recognition memory for words, and younger adults outperformed older adults on recognition memory for both modalities. This is supported by previous studies with the COLT, and in previous studies it has been suggested that recall abilities are lower for odors than for words due to the presence of an additional component of retrieval in recall when compared to recognition (Murphy et al., 1997). False positives were of interest because of their importance in dementia (Flanagan et al., 2016). Both young and old adults were more likely to make false positive errors in the recognition task for odors when compared to words. False positives in older adults on the Brief form of the COLT 3 suggest that older adults have difficulty specifying odors previously smelled. Future research might further explore false positives as a particular aspect of recognition memory.

Poorer performance was observed specifically in intrusion errors on the immediate recall trials. Older adults made more intrusion errors for both words and odors than younger adults (Figure 2). Both younger and older adults made more intrusion errors for odors than for words. Table 1 shows the number of intrusion errors for odors and words for older adults and younger adults. This could suggest a domain specific lower performance reflected in odor recall intrusions, but could also be indicative of greater variability in odor labels, since intrusions were defined as odor labels that did not match odor identification later in the test or acceptable substitutions for correct odor names. Thus, older adults could have poor performance in specifying and recalling one distinct label for odors over multiple trials. Future research is necessary to specify the exact nature of intrusion errors for odors compared to words, but the results of this study provide promising evidence that older adults showed a large number of odor recall intrusions or at the very least showed poor ability to specify odor labels over time. Past research has shown age-related changes in both odor identification (Murphy et al., 2002) and in consistency of odor labeling (Russell et al., 1993), and it has also been found that semantic factors, such as odor naming ability, are predictive of poor odor memory (Larsson and Bäckman, 1993, 1997; Lehrner, 1999). Thus it is theoretically sound to say that poor ability to semantically encode odor labels may contribute to the observed poor performance in odor recall and recognition, especially when considering the high number of intrusion errors in older adults for odors. Taken as a whole, this suggests that older adults show poor performance in consistently applying odor labels and that this impedes ability to store odor labels in working memory, recall these labels later, and differentiate between labels in a recognition task.

Existing tests used to identify preclinical AD are insufficient to determine whether a given individual will develop AD, thus efforts are keen to develop tests with better sensitivity and specificity or that add to a composite score with better sensitivity and specificity. Because olfactory tests with cognitive components show differential performance in populations at risk for AD (e.g., in individuals with the APOE ε4 allele), optimizing such tests for use in clinical testing is important. The majority of the research on preclinical testing with olfactory measures has been carried out with tests of odor identification and thus is limited in scope (Murphy, 2019). The current test adds testing of odor learning, episodic odor memory, odor recall and thus, may prove useful as a complement to odor identification. These different olfactory tasks will target different underlying neural substrates and thus may supply important differential information.

It is important to be mindful of past research into the neural correlates of memory for odors when considering the results of this study. Past research has found that the neural underpinnings of memory change in healthy aging. Imaging studies have reliably found that prefrontal areas are recruited by older adults as a compensatory mechanism in episodic memory (St-Laurent et al., 2011). For example, in encoding older adults show lower activity in the left prefrontal cortex and medial temporal lobe and in retrieval prefrontal cortex activation has been found to be bilateral in older adults, contrasting to younger adults, who show right lateralized activity (Haase et al., 2013). Thus, healthy aging older adults have been found to recruit different neural networks to compensate for the aging process (Haase et al., 2013). In olfactory memory, consistent involvement of the entorhinal cortex, piriform cortex, amygdala, hippocampus, and orbitofrontal cortex has been documented (Haase et al., 2013), and in olfactory working memory the orbitofrontal cortex has been found to be activated bilaterally (Dade et al., 2001). Since the Brief COLT requires engagement of encoding and retrieval processes, as well as working and recognition memory, it stands to reason that prefrontal and orbitofrontal, as well as medial temporal areas, would be engaged in this task. Thus, the Brief COLT may allow for a probe of neural functioning in medial temporal and prefrontal areas.

This study provided significant further evidence of poorer odor memory abilities in healthy aging. While this study was limited by a small sample size and the lack of direct comparisons between the brief and the full versions of the CVLT and the COLT in the same subjects in this first study, the fact that age effects similar to those in previous studies involving the COLT were observed in this study, particularly poorer performance in immediate odor recall, odor learning, false positives, and intrusion errors, lends credence to the idea that a brief measure of odor recall could be instrumental in probing neural functioning in areas implicated heavily in the aging process. The sample size prevented the testing of the potential effects of gender, a variable that will be of interest in future, larger studies. Further research could be critical in elucidating the nature of olfactory memory deficits in aging in order to further understand what changes we observe in healthy aging, which would be vital in detecting preclinical AD pathology. Future research will include a direct test of the brief and full-length forms of the COLT.

## Data Availability Statement

The datasets generated for this study are available on request to the corresponding author.

## Ethics Statement

The studies involving human participants were reviewed and approved by the San Diego State University Institutional Review Board. The patients/participants provided their written informed consent to participate in this study.

## Author Contributions

CF directed the data collection, performed the statistical analysis, and wrote the draft of the manuscript. CM contributed to the conception and design of the study and revised the manuscript.

## Conflict of Interest

The authors declare that the research was conducted in the absence of any commercial or financial relationships that could be construed as a potential conflict of interest.
